# Association of Shoe Cushioning Perception and Comfort With Injury Risk in Leisure‐Time Runners: A Secondary Analysis of a Randomised Trial

**DOI:** 10.1002/ejsc.70063

**Published:** 2025-10-22

**Authors:** Laurent Malisoux, Nicolas Delattre, Axel Urhausen, Cedric Morio, Daniel Theisen

**Affiliations:** ^1^ Physical Activity, Sport & Health Research Group Luxembourg Institute of Health Strassen Grand‐Duchy of Luxembourg; ^2^ Decathlon Sports Lab Movement Sciences Department Villeneuve d’Ascq France; ^3^ Sports Clinic Centre Hospitalier de Luxembourg Luxembourg Grand‐Duchy of Luxembourg; ^4^ Luxembourg Institute of Research in Orthopedics, Sports Medicine and Science Luxembourg Grand‐Duchy of Luxembourg; ^5^ ALAN—Maladies Rares Luxembourg Luxembourg Grand‐Duchy of Luxembourg

**Keywords:** comfort, cushioning, footwear, injury prevention, perception

## Abstract

The attenuation of impact forces was related to lower injury risk. However, runners cannot easily assess these biomechanics when testing new running shoes. Alternatively, the comfort filter has been suggested as a new paradigm relating running shoes and injury risk. This secondary analysis of a large randomised trial aimed to investigate whether the perception of cushioning and global appreciation of running shoes were associated with injury risk. Running exposure and injury data were prospectively collected in healthy recreational runners over 6 months. Study participants completed a questionnaire during follow‐up on shoe cushioning perception at the heel, ideal shoe cushioning level at the heel and global appreciation of the study shoe using numerical rating scales. Values were subsequently categorised in tertiles. A Cox proportional hazards model was used to estimate adjusted hazard rate ratios (HRs). The analysis includes 527 participants (35.3% females), and 60 of them reported at least one running‐related injury. Compared to the reference group (first tertile), participants with perception of moderate shoe cushioning (HR [95% CI] = 0.35 [0.19–0.66]) and those with perception of high shoe cushioning (HR = 0.24 [0.10–0.57]) had a lower injury risk. Similarly, participants with medium values (HR = 0.44 [0.25–0.78]) and highest values (HR = 0.33 [0.16–0.68]) for perceived‐ideal cushioning difference had a lower injury risk. Injury risk was also lower in participants with the highest global appreciation of the shoe (HR = 0.47 [0.26–0.85]). This study shows that greater perceived shoe cushioning and global appreciation are associated with lower injury risk, which suggests that cushioning perception, and potentially comfort filter, may be a valuable approach for shoe selection to prevent running‐related injuries.

**Trial Registration:**

ClinicalTrials.gov: NCT03115437, 11/04/2017.

## Highlights

- The comfort filter has been proposed as a new paradigm for the selection of running shoes to reduce injury risk, but no study has prospectively investigated this relationship.
- Greater perceived shoe cushioning and global appreciation of the shoe are associated with lower injury risk.
- Perceived cushioning, and potentially the comfort filter, may be valuable approaches for shoe selection to prevent running‐related injuries, although the validity of this paradigm deserves further research and the causal pathway has yet to be explained.

## 1 Introduction

Despite the evolution of running shoe technology over the last decades, there is no evidence that the incidence of running‐related injury has decreased (Nigg et al. 2015). Although this absence of detectable positive evolution in injury risk can be explained by many factors, such as a change in the running population over time, the large variability in reported injury incidence (Videbaek et al. 2015) and discrepancies in study design (e.g., data collection methods and injury definition), the scientific community generally agrees that the role of running shoe technology in injury prevention has probably been largely overrated (Malisoux and Theisen 2020) and that high‐quality RCTs are lacking (Napier and Willy 2018). Still, there is some evidence that the running shoe may influence injury risk (Fuller et al. 2017; Malisoux and Theisen 2020), and the question of how runners should be guided when buying new shoes remains unanswered.

Most running‐related injuries are overuse injuries (Malisoux et al. 2020) and result from repetitive loading of the musculoskeletal system (Bertelsen et al. 2017). Therefore, any shoe technology able to reduce the magnitude of loading may help preventing overuse injuries. Shoe cushioning systems were developed precisely to attenuate loads by redistributing them in time and space, the magnitude of cushioning effect being mainly determined by the shoe sole's material properties and geometry. The protective effect of shoe cushioning against running‐related injuries has recently been demonstrated in a large randomised trial including 800+ leisure‐time runners (Malisoux et al. 2020). The underlying mechanism includes a decrease in loading rate and an increase in time to impact peak of the high‐frequency signal in softer shoes (Malisoux, Gette, et al. 2021). However, the choice of appropriate running shoes remains extremely challenging for the runners as they cannot easily access standard values for the cushioning of footwear available on the market nor can they determine their personal optimal level of cushioning for injury prevention. Furthermore, shoe shops do not have the equipment and resources for accurate assessment of running biomechanics, especially impact forces and their relevant frequency components.

Alternatively, the comfort filter has previously been suggested as a new paradigm relating running shoes and injury risk. This paradigm proposes that runners should select a comfortable product according to this filter to reduce injury risk (Nigg et al. 2015). It assumes that runners can intuitively select a shoe that is biomechanically optimal for them based on perceived comfort. The main hypothesis is that impact forces, which have been related to injury risk (Malisoux, Gette, et al. 2021), induce ‘muscle tuning’ (i.e., activation) before initial contact of the foot with the ground. The muscle activation aims to minimise soft tissue vibration and might influence comfort perception and energy expenditure (Nigg 2001). Another hypothesis is that less comfortable shoes lead to a more repetitive kinematic pattern during running because they offer runners fewer options for comfortably executing the running movement task (Mohr et al. 2017). However, recent research could not confirm the link between comfort and coordination variability (Lindorfer et al. 2020).

There is no specific shoe feature associated with the comfort filter, although there are some indications that shoes with more cushioning are often perceived as the most comfortable (Dinato et al. 2015). Also, some studies have investigated the relationship between perceived shoe cushioning and biomechanical risk factors. For instance, perceived shoe cushioning was reported to highly correlate with ground reaction force parameters, including loading rate (Milani et al. 1997). Overall, it seems that perceived shoe cushioning and comfort may be used as a proxy of some biomechanical features, especially impact loading.

Nowadays, the comfort filter paradigm seems to be well accepted and to align with the real world experience as comfort and cushioning were found to be among the most commonly cited factors to influence running shoe selection in a recent systematic review (Fife et al. 2023). However, no study has prospectively investigated the relationship between subjective comfort, perceived cushioning and injury risk (Agresta et al. 2022). Therefore, the objective of this study was to investigate whether perception of cushioning and global appreciation of the running shoes were associated with injury risk in a cohort of 800+ leisure‐time runners followed up over 6 months. We hypothesised that runners with high perception of shoe cushioning, those with a larger difference between perceived and ideal cushioning levels and those with higher global appreciation of the running shoes would have lower injury risk.

## 2 Materials and Methods

### 2.1 Study Design

This is a secondary analysis of a participant and assessor‐blinded randomised trial comparing two running shoe prototypes with different cushioning properties (Malisoux et al. 2017). The intervention lasted 6 months, during which running exposure and injuries were self‐reported in a dedicated electronic platform (Malisoux et al. 2013a; Theisen et al. 2014). The trial was registered at ClinicalTrials.gov (NCT03115437, 11/04/2017) before recruitment. The study was approved by the National Ethics Committee for Research (Ref: 201701/02 v1.1). All volunteers received a full description of the study and provided written informed consent for participation. Reporting of the study followed the CONSORT (Consolidated Standards of Reporting Trials) statement (Moher et al. 2010).

### 2.2 Patient Involvement Statement

Study participants were not involved in the design, conduct, interpretation or translation of the current research.

### 2.3 Participants

The parent trial included leisure‐time runners, regardless of running experience, fitness level or body mass. Leisure‐time runners were recruited from September 2017 to January 2018 from Luxembourg and the neighbouring regions. Inclusion criteria were being in good health, aged 18–65 years and capable of performing 15 min of consecutive running were included in the study. Exclusion criteria were any running‐impeding injury over the previous month, medical contraindication to perform running activity, prior (< 12 months) lower limb or back surgery and use of orthopaedic insoles for running.

### 2.4 Baseline Evaluations and Follow‐Up

Volunteers completed an online questionnaire regarding running experience and previous injury. During the subsequent visit to the laboratory, height, body mass, fat mass proportion (Tanita SC‐240 MA) and leg length (Malisoux et al. 2017) were measured. Participants randomly received one of two running shoe versions specifically designed for the parent trial (Malisoux et al. 2017, 2020), differing only in their cushioning properties (i.e., high vs. low) (Malisoux, Delattre, et al. 2021; Malisoux et al. 2023).

Over the following 6 months, the participants self‐reported all their sports activities, running session characteristics (e.g., duration, distance and subjectively perceived intensity) and any adverse events (injuries, pain and illnesses) during any given session on a dedicated electronic platform (Malisoux et al. 2013a, 2013b). Among others, mandatory information for each running session included the shoe pair used and whether the participant had experienced any pain during the session.

### 2.5 Main Exposure

A short questionnaire was developed based on (Delattre and Cariou 2017) and submitted to the participants during the intervention at a randomly determined moment to collect feedback on shoe cushioning perception and global appreciation. The questionnaire included three questions on (1) the perception of study shoe cushioning at the heel, (2) the participant's desired ideal level of shoe cushioning at the heel and (3) the participant's global appreciation of the study shoe (except for the aesthetic properties). A numerical rating scale was used for each question (score from min. 1 to max. 9). The difference between shoe cushioning perception (Question 1) and ideal shoe cushioning level (Question 2) was computed to reflect the perceived‐ideal cushioning difference.

### 2.6 Primary Outcome

The primary outcome was the first running‐related injury occurring during the 6‐month follow‐up, defined as a running‐related musculoskeletal pain in either of the two lower limbs that causes a restriction or stoppage of running (distance, speed or duration) for at least 7 days (adapted from Yamato et al. 2015). The research team checked all injury data for coherence and contacted the participants for a final check of all recordings.

### 2.7 Statistical Analyses

All analyses were performed using STATA/SE version 15. Data pertaining to demographic, training and shoe perception characteristics were presented as counts and percentages for dichotomous variables and as means ± standard deviations (SD) or medians and interquartile ranges for normally or abnormally distributed continuous variables, respectively. Normality was assessed using histograms. Analysis of variance (ANOVA) was used to compare the shoe perception characteristics between the hard and the soft shoe groups. Spearman correlation coefficients were computed to describe the relationship between the four shoe perception characteristics.

The association between shoe perception variables and injury risk was investigated using competing‐risks proportional hazards regression models, which estimate the subdistribution hazard (subhazard rate ratio, SHR; Fine and Gray 1999). Time at risk was defined as hours of running practice cumulated from baseline evaluation date to date of injury or censoring. The assumption of proportional hazards was evaluated using log‐minus‐log plots and Schoenfeld residuals. All shoe perception characteristics were categorised in tertiles as we assumed that their relationship with injury risk is not linear. The first tertile was defined as the reference group. Model 1 presents the crude estimates of SHR and their 95% confidence interval (CI) for each exposure separately. Model 2 adjusted for potential confounders. Model 3 is model 2 with mutual adjustment for all shoe perception variables (except perceived‐ideal cushioning difference i.e., computed from 2 other variables). Model 1–3 were run again using the second tertile to investigate difference in injury risk between the third and second tertile. A directed acyclic graph (DAG) representing the causal pathways between shoe cushioning perception and running‐related injury is presented in Supporting Information S1: Material S1. Statistical significance was set at *p* < 0.05, but we place emphasis on the range of plausible values of associations as indicated by CIs (Amrhein et al. 2019).

## 3 Results

### 3.1 Participants

Eight hundred seventy‐four volunteers fulfilled the inclusion criteria and were randomly allocated to one of the study groups in the parent study. Three hundred forty‐seven participants were excluded from the current analysis because they did not fill in the questionnaire on shoe perception (*n* = 321), did not upload any training data (*n* = 19), were diagnosed with arthrosis during follow‐up (*n* = 3), reported untrustworthy training data (*n* = 2), used orthopaedic insoles (*n* = 1) or had another health issue (*n* = 1). A flowchart is presented in Supporting Information S1: Material S2.

**FIGURE 1:**
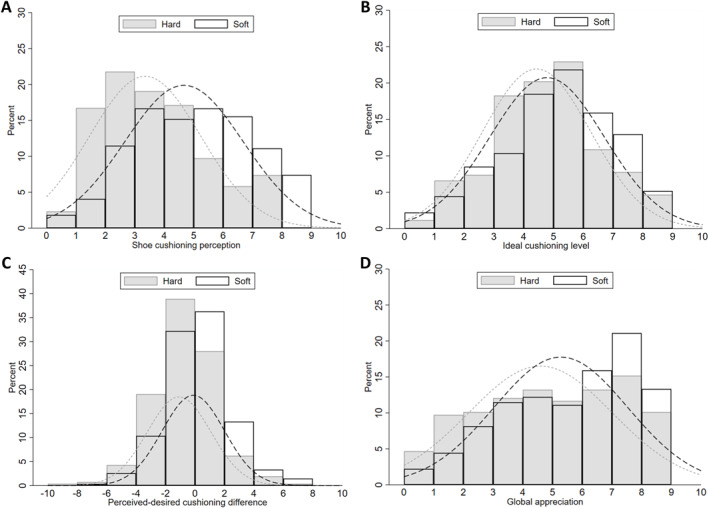
Distribution of the shoe perception characteristics (scores from min. 1 to max. 9) per shoe group (hard and soft shoe versions, respectively): Shoe cushioning perception (A), ideal shoe cushioning (B), perceived‐desired cushioning difference (C) and global appreciation (D).

Table 1 presents the descriptive statistics of the 527 participants analysed. Age was 41.1 years (SD 10.2), 35.3% were females and body mass index was 24.3 (SD 3.1). The participants reported 17,626 h of running over the follow‐up, for a total of 172,307 km, of which 165,181 km (96%) with the study shoes (97.6% of the 18,815 running sessions reported). On average, the participants performed 29 ± 20 running sessions with the study shoes before completing the questionnaire on cushioning perception, which corresponds to 261 ± 249 km (or 26.7 ± 24.0 h of running). The ANOVA revealed that participants who received the soft shoe version reported greater perceived cushioning at the heel (mean difference: 1.3 ± 0.2 a.u. and *p* < 0.001), slightly higher desired ideal cushioning level (0.4 ± 0.2 a.u. and *p* = 0.032), which translated into a larger perceived‐ideal cushioning difference (0.97 ± 0.2 a.u. and *p* < 0.001), as well as a greater global appreciation of the shoe (0.7 ± 0.2 a.u. and *p* = 0.001), compared to those who received the hard shoe version (Figure 1).

**TABLE 1:** Descriptive statistics of the participants.

Characteristics	Unit/qualifier	All participants *n* = 527	Soft shoe *n* = 270	Hard shoe *n* = 257
Participants' characteristics				
Age	Years	41.1 ± 10.2	40.5 ± 10.4	41.6 ± 9.9
Sex	Male	341 (64.7%)	180 (66.7%)	161 (62.6%)
	Female	186 (35.3%)	90 (33.3%)	96 (37.4%)
Height	cm	174 ± 9	175 ± 9	174 ± 9
Body mass	kg	74.3 ± 12.6	74.5 ± 12.9	74.0 ± 12.3
Body mass index	kg.m^−2^	24.3 ± 3.1	24.3 ± 3.3	24.3 ± 2.9
Proportion of fat mass	%	22.5 ± 7.3	22.1 ± 7.1	22.9 ± 7.6
Previous injury	No	443 (84.1%)	229 (84.8%)	214 (83.3%)
	Yes	84 (15.9%)	41 (15.2%)	43 (16.7%)
Running experience	Years	6 [3 to 14]	6 [3 to 15]	5 [3 to 12]
Regularity (last 12 months)	Months	12 [6 to 12]	12 [6 to 12]	12 [6 to 12]
Running participation (follow‐up)				
Running frequency	Sessions. week^−1^	1.4 [1.0 to 2.0]	1.5 [1.0 to 2.0]	1.4 [1.0 to 2.0]
Mean session duration	Minutes	51 [41 to 61]	52 [42 to 62]	50 [41 to 61]
Mean session distance	km	8.2 [6.2 to 10.1]	8.3 [6.5 to 10.3]	8.1 [5.9 to 9.9]
Mean session intensity	a.u. [1 to 10]	3.9 [3.9 to 4.5]	3.9 [3.9 to 4.6]	3.8 [3.2 to 4.4]
Mean speed	km.h^−1^	9.7 [8.8 to 10.7]	9.8 [8.9 to 10.7]	9.7 [8.6 to 10.5]
Shoe perception				
Shoe cushioning perception	a.u. [1 to 9]	4 [2 to 5]	5 [3 to 6]	3 [2 to 5]
Ideal shoe cushioning level	a.u. [1 to 9]	5 [3 to 6]	5 [4 to 6]	5 [3 to 5]
Perceived‐ideal cushioning difference	a.u. [1 to 9]	0 [−2 to 1]	0 [−1 to 1]	−1 [−2 to 0]
Global appreciation	a.u. [1 to 9]	5 [3 to 7]	6 [4 to 7]	5 [3 to 7]

*Note:* Normally and abnormally distributed variables are presented as mean ± standard deviation and median [interquartile range], respectively.

Abbreviation: a.u., arbitrary unit.

Global appreciation of the shoe was positively correlated with both shoe cushioning perception (*r* = 0.24 and *p* < 0.001) and perceived‐ideal cushioning difference (*r* = 0.22 and *p* < 0.001), which were also correlated together (*r* = 0.57 and *p* < 0.001; Supporting Information S1: Material S3). Yet, these correlations were weak to moderate.

### 3.2 Injuries

In total, 119 participants reported at least one injury during follow‐up. The first injury reported in the electronic system met the running‐related injury definition for 60 of the participants, and it was defined as a competing injury (e.g., ankle sprain when playing soccer and bike accident) for 59 participants. Running‐related injury incidence was 3.4 injuries per 1000 h of running (95% CI: 2.6–4.4). The characteristics of self‐reported running‐related injuries are presented in Table 2.

**TABLE 2:** Characteristics of self‐reported running‐related injuries (*n* = 60).

	All participants	
	*N*	%
Injury location		
Lower back	2	3.3
Buttock/Pelvis	2	3.3
Hip/Groin	3	5.0
Thigh	3	5.0
Knee	10	16.7
Lower leg	13	21.7
Ankle	16	26.7
Foot	11	18.3
Injury type		
Tendon	25	41.6
Muscle	12	20.0
Capsules and ligaments	10	16.7
Bone structures	0	0.0
Other joint structure	3	5.0
Other overuse injuries	10	16.7
Injury severity		
Moderate (8–28 days)	45	75.0
Major (> 28 days)	15	25.0
Context		
Training	56	93.3
Competition	4	6.7

### 3.3 Injury Risk

**FIGURE 2:**
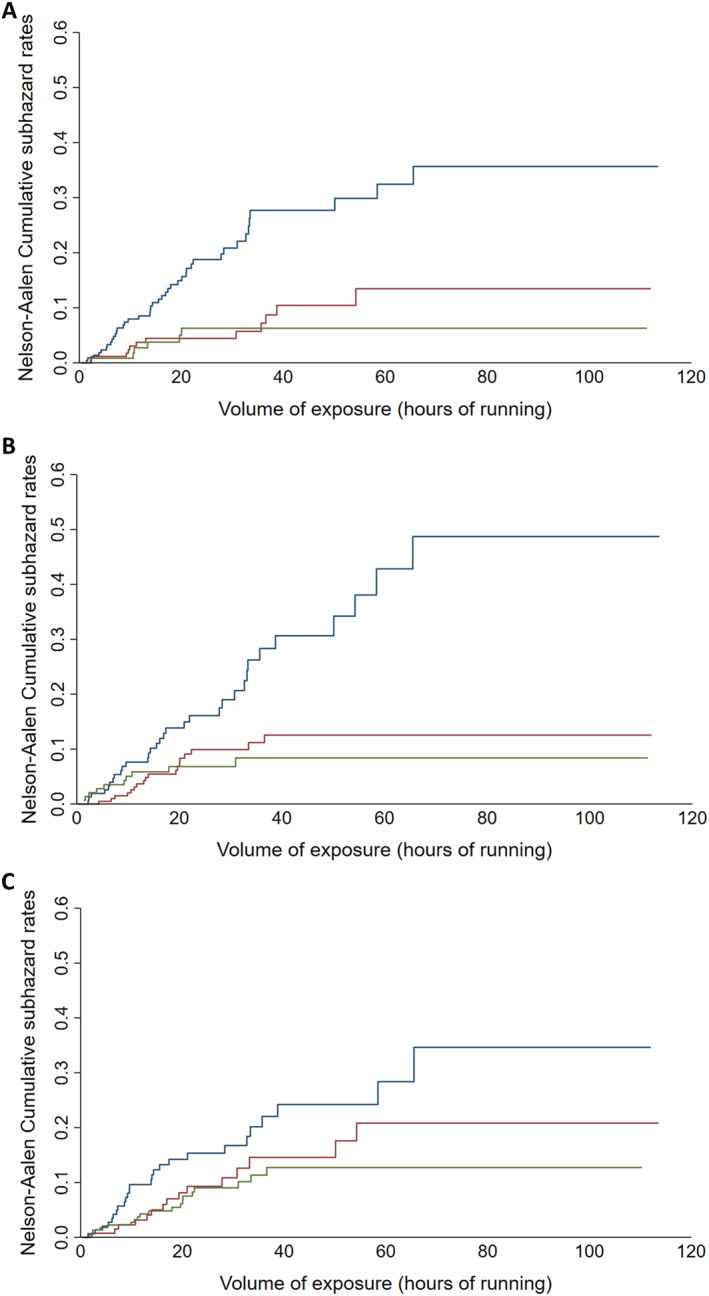
Cumulative incidence functions for running‐related injury according to shoe cushioning perception (A), perceived‐ideal cushioning difference (B) and global appreciation (C). The blue line is the first tertile (reference group), the red line is the middle tertile and the green line is the third tertile.

Table 3 presents the estimates of the SHR for each shoe perception characteristic. In the crude analysis (Model 1), participants with perception of moderate shoe cushioning (i.e., from > 3 to < 6 a.u., second tertile) and those with perception of high shoe cushioning (i.e., ≥ 6 a.u., third tertile) had a lower risk of running‐related injury than the reference group (i.e., first tertile, Figure 2A). Similarly, participants with medium values (i.e., from > −2 to ≤ 0 a.u.) and those with the highest values for perceived‐ideal cushioning difference (i.e., > 0 a.u.) had a lower running‐related injury risk (Figure 2B). Participants with the highest global appreciation of the shoe (i.e., ≥ 6 a.u) had a lower injury risk (Figure 2C). Adjustment for potential confounders (i.e., previous injury and body mass, Model 2) marginally influenced the estimates, thus confirming the main findings from Model 1. Mutual adjustment for all shoe perception variables (Model 3) also confirmed the lower injury risk observed in those with moderate and high perception of shoe cushioning, whereas global appreciation of the shoe was no longer associated with injury risk.

**TABLE 3:** Association between shoe perception characteristics and injury risk (*n* = 527).

Variables	Injuries (participants)	Model 1	Model 2	Model 3
Shoe cushioning perception				
Tertile 1	42 (220)	Ref	Ref	Ref
Tertile 2	12 (181)	0.34 [0.18; 0.64]; ***0.001***	0.35 [0.19; 0.66]; ***0.001***	0.36 [0.18; 0.70]; ***0.002***
Tertile 3	6 (126)	0.24 [0.10; 0.56]; ***0.001***	0.24 [0.10; 0.57]; ***0.001***	0.23 [0.10; 0.53]; ***0.001***
Ideal shoe cushioning level				
Tertile 1	17 (133)	Ref	Ref	Ref
Tertile 2	26 (242)	0.87 [0.47; 1.60]; *0.648*	0.89 [0.48; 1.64]; *0.703*	1.05 [0.56; 1.97]; *0.869*
Tertile 3	17 (152)	0.95 [0.48; 1.86]; *0.875*	0.97 [0.49; 1.90]; *0.922*	1.50 [0.75; 3.00]; *0.255*
Perceived‐ideal cushioning diff.				
Tertile 1	32 (166)	Ref	Ref	—
Tertile 2	18 (212)	0.41 [0.23; 0.72]; ***0.002***	0.44 [0.25; 0.78]; ***0.005***	—
Tertile 3	10 (149)	0.32 [0.16; 0.66]; ***0.002***	0.33 [0.16; 0.68]; ***0.003***	—
Global appreciation				
Tertile 1	26 (151)	Ref	Ref	Ref
Tertile 2	15 (141)	0.59 [0.31; 1.11]; *0.101*	0.59 [0.31; 1.13]; *0.114*	0.77 [0.39; 1.51]; *0.440*
Tertile 3	19 (235)	0.46 [0.25; 0.83]; ***0.010***	0.47 [0.26; 0.85]; ***0.012***	0.60 [0.33; 1.09]; *0.092*

*Note:* Running exposure: 17,626 h; 60 running‐related injuries; Values are subhazard rate ratio [95% confidence interval]; *p* values are presented in *Italics*; *p* values < 0.05 are presented in bold. Model 1 is the unadjusted model. Model 2 = Model 1 adjusted for previous injury and body mass. Model 3 = Model 2 mutually adjusted for all shoe perception characteristics.

No difference was observed between the second and the third tertile for any of the shoe perception characteristic, when the second tertile was used as a reference (Supporting Information S1: Material S4). The latter finding suggests that the relationship with injury risk is not linear. Sensitivity analyses using shoe perception characteristics as continuous variables were in line with the main findings, except that global appreciation was no longer significantly associated (*p* = 0.058) with injury risk in Model 2 (Supporting Information S1: Material S5).

The estimates of the SHR for each shoe perception characteristic, stratified by shoe version (Model 4), are presented in Supporting Information S1: Material S6. Although some of the previous observations are confirmed, others are not, most probably because of a lack of statistical power.

## 4 Discussion

The objective of this study was to investigate whether the perception of cushioning and global appreciation of the running shoes were associated with injury risk in a large cohort of leisure‐time runners. This study is the first to provide scientific evidence, based on prospective data collection, that cushioning perception, perceived‐ideal cushioning difference and global appreciation of the shoe (except for the aesthetic properties) are related to injury risk in recreational runners. These findings suggest that perceived cushioning, and potentially the comfort filter, may be valuable approaches for shoe selection to prevent running‐related injuries. Nevertheless, the validity of this paradigm deserves further research, especially using a prospective design, and the causal pathway relating cushioning perception, comfort and injury development has yet to be explained.

### 4.1 Paradigm Shift

Over the last decade, the role of footwear technology in injury prevention has been questioned (Agresta et al. 2022; Malisoux and Theisen 2020; Theisen et al. 2016), especially because the spectacular technological development of the running shoe has not been parallelled with a noticeable decrease in injury incidence (Nigg et al. 2015). However, *absence of evidence is not evidence of absence*, and several reasons may explain that why such a trend has not been detected: (1) there is no monitoring system that collects data over time, allowing an accurate assessment of the progression of injury incidence in a given population of runners, (2) the overall population of runners may have changed over time and could include a much higher proportion of more injury‐prone novice runners (Videbaek et al. 2015) and (3) the definition of running injury varies widely across studies (from symptoms of pain or discomfort to complaints that required medical attention) (Lacey et al. 2024). In short, the available data does not allow direct comparisons between different study periods or methodologies.

Nevertheless, in the absence of strong scientific evidence for recommendations on specific shoe features (e.g., cushioning, heel‐to‐toe drop and motion control system) for injury prevention, it has been proposed to replace the previous paradigms of ‘cushioning’ and ‘pronation’ with the two new paradigms of ‘preferred movement path’ and ‘comfort filter’ (Nigg et al. 2015). The latter suggests that subjective comfort is the most important factor for selecting running shoes to reduce injury risk. The authors also stated that substantial new research is needed regarding the relation between comfort filter and running performance or injuries. Although this paradigm was suggested a decade ago, the present study is the first to investigate the relationship between comfort and more specifically the perception of cushioning and the risk of injury.

### 4.2 Plausible Explanations

The present study demonstrates that injury risk was lower in runners with greater perception of shoe cushioning, those with greater difference between cushioning perception and expectation (i.e., desired ideal level of cushioning) and/or those with greater global appreciation of the shoe. Of course, one may assume that, if participants appreciated their shoes, they may have rated specific shoe attributes highly. This assumption was confirmed by a positive correlation between perceived cushioning and global appreciation (Supporting Information S1: Material S3), although the correlation was weak. Globally, after mutual adjustment for all shoe perception variables (Model 3, Table 3), perceived cushioning appeared to be the most important feature for injury prevention. However, other shoe perception characteristics may no longer be associated with the outcome because most of the variance is explained by perceived cushioning or a lack of statistical power. Therefore, our main model is Model 2 as it shows which characteristics are related to injury risk, and Model 3 only indicates that shoe cushioning perception was more relevant than global appreciation for injury risk in our study. Actually, comfort is multifactorial and poorly defined, with both cushioning and fit shown to affect comfort (Fife et al. 2023). To date, there is no standardised method to assess comfort and shoe attributes perception other than asking the runner to rank or select shoes based on individual preference (Agresta et al. 2022). A running shoe comfort assessment tool (RUN‐CAT) has recently been developed and confirms that shoe cushioning is one of the key components of comfort (Bishop et al. 2020).

Given the key role of cushioning for comfort perception, we also investigated whether a discrepancy between expected and actual perception of shoe cushioning may be related to injury risk. Indeed, every runner is unique, with their own running biomechanics. Consequently, the runner is the only one who knows what type of cushioning is best suited to their needs. We speculated that a low perceived‐ideal cushioning difference is associated with injury risk. This study confirmed that runners in the first tertile (values ≤ −2 a.u.) had more than twice the risk of injury compared to the second tertile (values > −2 to 0 a.u.), whereas those in the third tertile (values > 0 a.u.) showed no additional benefits (Supporting Information S1: Material S4). The latter finding suggests that the relationship with injury risk is not linear. Therefore, based on these results, the comfort filter may have particular relevance to running injuries for negative perceived‐ideal cushioning differences, thus acting as some sort of high‐pass filter in terms of injury prevention.

One hypothesis about the possible connection between shoe comfort and injury risk is that tissue vibrations resulting from impact forces must be counteracted by muscle activation, which requires higher energy expenditure and negatively impacts comfort (Nigg 2001). A second hypothesis stems from studies which found differences in coordination variability between healthy and injured runners (Heiderscheit et al. 2002) and suggests that biomechanical variability may also be involved in the development of overuse injury (Hamill et al. 2012). As reduced variability in running kinematics was associated with lower footwear comfort (Mohr et al. 2017), it was therefore also speculated that a less comfortable shoe may offer fewer solutions for a runner to execute the running movement comfortably, leading to a more repetitive kinematic pattern.

### 4.3 Clinical Implications

The relationship between running shoes properties and injury risk is complex and lacks evidence from high‐quality research. Still, health professionals make recommendations to patients about which running shoes to select to reduce injury risk. Furthermore, both clinicians (Dhillon et al. 2020) and runners (Saragiotto et al. 2014) have predefined ideas about risk factors, including running shoes, whereas these risk factors are not always scientifically supported (Wolthon et al. 2020). Some of the challenges health professionals face when recommending running shoes are that (1) injuries are multifactorial and the shoe may (or may not) be one of the many factors involved in the causal pathway (moderation effect), (2) most of the mechanisms relating running shoes to injury risk are not well‐understood and (3) every runner is unique and may react differently to a given shoe feature (Malisoux and Theisen 2020; Theisen et al. 2016). In this respect, science can provide some general guidelines, but the final decision will always be individual and should preferably be based on correct and unbiased information. Biomechanical analysis of the runner's technique may offer relevant information, but such analyses are resource‐intensive and require high expertise for data interpretation. Above all, translating any biomechanical observation into recommendations about running shoe characteristics remains challenging and highly speculative. This brings us back to the idea that the most relevant aspect to reduce injury risk may well be choosing the most comfortable shoe (Nigg et al. 2015). Instead of speculating from running biomechanics to the appropriate shoe features, comparing comfort and shoe features perception while running in different shoe models may represent a more effective approach to preventing running injuries. However, it should be noted that the present findings are based on a heterogeneous sample of recreational runners using neutral cushioned shoes with minimal structural differences. Therefore, generalisation to other populations, such as elite athletes or those using shoes with special features (e.g., pronation support and carbon plate), should be done with caution.

### 4.4 Strengths and Limitations

This is the first prospective study investigating the association of cushioning perception and global appreciation of the running shoes with running‐related injury risk. The analysis includes a large sample of recreational runners (*n* = 527) who used the study shoes for 97.6% of their running sessions over 6 months. However, this study also has some limitations. First, a part of the study participants did not complete the comfort questionnaire, most probably because they dropped out before they received it, which could have led to selection bias. Some differences were observed between the participants included in this study and those from the parent trial that were excluded (Supporting Information S1: Material S7). Notably, excluded participants were younger (mean difference: 1.6 years) and lighter (2.5 kg), they practiced running less regularly (0.1 session/week), they had shorter mean session duration (2 min) and distances (0.5 km) and they reported lower intensities (0.4 a.u. on the numerical rating scale) compared to the included participants. These differences, although statistically significant (because of the large sample size), were marginal, and none of these cofactors has been directly associated with injury risk in previous analyses. Still, the reasons for dropping out are probably not completely random as it is likely that predominantly participants with low global appreciation of the shoes withdrew from the study. Second, the questionnaire was not administered at baseline or early on in the follow‐up. Consequently, some participants may have sustained an injury before completing the questionnaire and therefore, rated their shoes more negatively, potentially inducing reverse causation. Hence, further prospective studies with assessment of cushioning perception and global appreciation at baseline and at regular time points are needed to investigate a possible causal pathway.

### 4.5 Research Perspectives

The present study clearly falls into causal inference, which aims to investigate and estimate the effect of an exposure (i.e., high perception of cushioning and/or high global appreciation of the running shoes) on injury development in a given population (Ito et al. 2025). Although a randomised controlled trial is considered as the method of choice for causal research questions, investigating the comfort filter paradigm and shoe attributes perception with such a study design does not seem realistic. Indeed, runners may not accept using uncomfortable running shoes for several months, and they may be strongly biased if they understand that they were allocated to the study arm with suboptimal shoes with regards to comfort. Furthermore, such trial may be considered unethical. Although there is still a mistaken belief that causal inference is not possible in observational research, certain research questions can only be answered using observational studies (Hernán and Robins 2020). A transparent reporting of a clear causal framework (e.g., in the form of a directed acyclic graph, Supporting Information S1: Material S1) is essential to evaluate whether results can be interpreted causally (Tennant et al. 2021). In any case, researchers should state their causal research aims, discuss whether the assumptions are likely to be met and make associational interpretations if interpreting results causally is deemed inappropriate (Ito et al. 2025).

Some of the limitations of the present study can be improved by design (see above). Future research on running injuries may collect information on shoe attributes perception at several time points, including after a few hours of running, to investigate the effect of both absolute attributes perception and change in perception due to shoe wear and tear during follow‐up on injury risk, while properly adjusting for confounders and minimising the amount of missing data (Malisoux et al. 2024). Furthermore, some research suggested that forefoot cushioning is more strongly related to comfort than heel cushioning, and consequently, its association with injury risk deserves to be investigated (Bishop et al. 2020).

## 5 Conclusion

The present study showed that greater perceived shoe cushioning and global appreciation of the shoe are associated with lower risk of running‐related injury, and that perceived shoe cushioning appeared to be the most important feature for injury prevention. Consequently, the study findings are consistent with the comfort filter paradigm and suggest that perceived shoe cushioning, and comfort, may be a valuable approach for shoe selection to prevent running‐related injuries. However, causality cannot be inferred, and further studies are needed to disentangle the relationship between running biomechanics, shoe perception and injury risk.

## Author Contributions

L.M., N.D., A.U., C.M. and D.T. contributed sufficiently to the manuscript to justify authorship. L.M., N.D. and D.T. conceptualised the project and defined the methodology. L.M. and D.T. collected the data. L.M. conducted the analysis. All authors were involved in the interpretation of the results. L.M. drafted the original manuscript and all other authors provided significant feedback and comments in refining the final manuscript. All authors approved the final manuscript. All authors agree to be accountable for the content of the work.

## Ethics Statement

The study was approved by the National Ethics Committee for Research in Luxembourg (Ref: 201701/02 v1.1).

## Consent

All volunteers received a full protocol description and provided written informed consent for participation.

## Permission to Reproduce Material From Other Sources

The authors have nothing to report.

## Conflicts of Interest

N.D. and C.M. were employed at Decathlon. Decathlon also provided the standard running shoes. A research partnership agreement was signed between Decathlon and the LIH.

## Supporting information


Supporting Information S1

Supporting Information S2

## Data Availability

Data are available upon request. Individual values for each variable related to perception and comfort can be requested for research purpose only via email to the corresponding author (Dr. Laurent Malisoux).

## References

[ejsc70063-bib-0001] Agresta, C. , C. Giacomazzi , M. Harrast , and J. Zendler . 2022. “Running Injury Paradigms and Their Influence on Footwear Design Features and Runner Assessment Methods: A Focused Review to Advance Evidence‐Based Practice for Running Medicine Clinicians.” Frontiers in Sports and Active Living 4: 815675. 10.3389/fspor.2022.815675. 35356094 PMC8959543

[ejsc70063-bib-0002] Amrhein, V. , S. Greenland , and B. McShane . 2019. “Scientists Rise up Against Statistical Significance.” Nature 567, no. 7748: 305–307. 10.1038/d41586-019-00857-9. 30894741

[ejsc70063-bib-0003] Bertelsen, M. L. , A. Hulme , J. Petersen , et al. 2017. “A Framework for the Etiology of Running‐Related Injuries.” Scandinavian Journal of Medicine & Science in Sports 27, no. 11: 1170–1180. 10.1111/sms.12883. 28329441

[ejsc70063-bib-0004] Bishop, C. , J. D. Buckley , A. E. Esterman , and J. B. Arnold . 2020. “The Running Shoe Comfort Assessment Tool (RUN‐CAT): Development and Evaluation of a New Multi‐Item Assessment Tool for Evaluating the Comfort of Running Footwear.” Journal of Sports Sciences 38, no. 18: 2100–2107. 10.1080/02640414.2020.1773613. 32508250

[ejsc70063-bib-0005] Delattre, N. , and A. Cariou . 2017. “Women Perception of Shoe Cushioning as a Function of Mechanical Properties of Footwear Using a Sensory Trained Panel Method.” Footwear Science 10, no. 1: 11–19. 10.1080/19424280.2017.1403973.

[ejsc70063-bib-0006] Dhillon, G. K. , M. A. Hunt , A. L. Reid , and J.‐F. Esculier . 2020. “What Are the Perceptions of Runners and Healthcare Professionals on Footwear and Running Injury Risk?” BMJ Open Sport & Exercise Medicine 6, no. 1: e000767. 10.1136/bmjsem-2020-000767. PMC7328975 32626599

[ejsc70063-bib-0007] Dinato, R. C. , A. P. Ribeiro , M. K. Butugan , I. L. Pereira , A. N. Onodera , and I. C. Sacco . 2015. “Biomechanical Variables and Perception of Comfort in Running Shoes With Different Cushioning Technologies.” Journal of Science and Medicine in Sport 18, no. 1: 93–97. 10.1016/j.jsams.2013.12.003. 24444754

[ejsc70063-bib-0008] Fife, A. , C. Ramsey , J.‐F. Esculier , and K. Hébert‐Losier . 2023. “How Do Road Runners Select Their Shoes? A Systematic Review.” Footwear Science 15, no. 2: 103–112. 10.1080/19424280.2023.2180543.

[ejsc70063-bib-0009] Fine, J. P. , and R. J. Gray . 1999. “A Proportional Hazards Model for the Subdistribution of a Competing Risk.” Journal of the American Statistical Association 94, no. 446: 496–509. 10.1080/01621459.1999.10474144.

[ejsc70063-bib-0010] Fuller, J. T. , D. Thewlis , J. D. Buckley , N. A. Brown , J. Hamill , and M. D. Tsiros . 2017. “Body Mass and Weekly Training Distance Influence the Pain and Injuries Experienced by Runners Using Minimalist Shoes: A Randomized Controlled Trial.” American Journal of Sports Medicine 45, no. 5: 1162–1170. 10.1177/0363546516682497. 28129518

[ejsc70063-bib-0011] Hamill, J. , C. Palmer , and R. E. Van Emmerik . 2012. “Coordinative Variability and Overuse Injury.” Sports Medicine, Arthroscopy, Rehabilitation, Therapy and Technology 4: 1–9. 10.1186/1758-2555-4-45. PMC3536567 23186012

[ejsc70063-bib-0012] Heiderscheit, B. C. , J. Hamill , and R. E. van Emmerik . 2002. “Variability of Stride Characteristics and Joint Coordination Among Individuals With Unilateral Patellofemoral Pain.” Journal of Applied Biomechanics 18, no. 2: 110–121. 10.1123/jab.18.2.110.

[ejsc70063-bib-0013] Hernán, M. A. , and J. M. Robins . 2020. Causal Inference: What if. Chapman & Hall/CRC. https://miguelhernan.org/whatifbookhttps://www.routledge.com/Causal‐Inference‐What‐If/Hernan‐Robins/p/book/9781420076165?srsltid=AfmBOoqpb6Z7WCO6AJEC7ZMJbdm‐Dr_t22RCm1PXJl‐eW5BgQY1Fn2ss.

[ejsc70063-bib-0014] Ito, C. , L. Al‐Hassany , T. Kurth , and T. Glatz . 2025. “Distinguishing Description, Prediction, and Causal Inference: A Primer on Improving Congruence Between Research Questions and Methods.” Neurology 104, no. 4: e210171. 10.1212/WNL.0000000000210171. 39899793

[ejsc70063-bib-0015] Lacey, A. , E. Whyte , S. Dillon , S. O’Connor , A. Burke , and K. Moran . 2024. “Definitions and Surveillance Methods of Running‐Related Injuries: A Scoping Review.” European Journal of Sport Science 24, no. 7: 950–963. 10.1002/ejsc.12123. 38956793 PMC11235823

[ejsc70063-bib-0016] Lindorfer, J. , J. Kroll , and H. Schwameder . 2020. “Does Enhanced Footwear Comfort Affect Oxygen Consumption and Running Biomechanics?” European Journal of Sport Science 20, no. 4: 468–476. 10.1080/17461391.2019.1640288. 31282808

[ejsc70063-bib-0017] Malisoux, L. , N. Delattre , C. Meyer , P. Gette , A. Urhausen , and D. Theisen . 2021. “Effect of Shoe Cushioning on Landing Impact Forces and Spatiotemporal Parameters During Running: Results From a Randomized Trial Including 800+ Recreational Runners.” European Journal of Sport Science 21, no. 7: 985–993. 10.1080/17461391.2020.1809713. 32781913

[ejsc70063-bib-0018] Malisoux, L. , N. Delattre , A. Urhausen , and D. Theisen . 2017. “Shoe Cushioning, Body Mass and Running Biomechanics as Risk Factors for Running Injury: A Study Protocol for a Randomised Controlled Trial.” BMJ Open 7, no. 8: e017379. 10.1136/bmjopen-2017-017379. PMC5724138 28827268

[ejsc70063-bib-0019] Malisoux, L. , N. Delattre , A. Urhausen , and D. Theisen . 2020. “Shoe Cushioning Influences the Running Injury Risk According to Body Mass: A Randomized Controlled Trial Involving 848 Recreational Runners.” American Journal of Sports Medicine 48, no. 2: 473–480. 10.1177/0363546519892578. 31877062

[ejsc70063-bib-0020] Malisoux, L. , A. Frisch , A. Urhausen , R. Seil , and D. Theisen . 2013a. “Injury Incidence in a Sports School During a 3‐Year Follow‐up.” Knee Surgery, Sports Traumatology, Arthroscopy 21, no. 12: 2895–2900. 10.1007/s00167-012-2185-1. 22968623

[ejsc70063-bib-0021] Malisoux, L. , A. Frisch , A. Urhausen , R. Seil , and D. Theisen . 2013b. “Monitoring of Sport Participation and Injury Risk in Young Athletes.” Journal of Science and Medicine in Sport 16, no. 6: 504–508. 10.1016/j.jsams.2013.01.008. 23481535

[ejsc70063-bib-0022] Malisoux, L. , P. Gette , A. Backes , N. Delattre , J. Cabri , and D. Theisen . 2021. “Relevance of Frequency‐Domain Analyses to Relate Shoe Cushioning, Ground Impact Forces and Running Injury Risk: A Secondary Analysis of a Randomized Trial With 800+ Recreational Runners [Clinical Trial].” Frontiers in Sports and Active Living 3, no. 320: 744658. 10.3389/fspor.2021.744658. 34859204 PMC8632264

[ejsc70063-bib-0023] Malisoux, L. , P. Gette , A. Backes , N. Delattre , and D. Theisen . 2023. “Lower Impact Forces but Greater Burden for the Musculoskeletal System in Running Shoes With Greater Cushioning Stiffness.” European Journal of Sport Science 23, no. 2: 210–220. 10.1080/17461391.2021.2023655. 35014593

[ejsc70063-bib-0024] Malisoux, L. , and D. Theisen . 2020. “Can the ‘Appropriate’ Footwear Prevent Injury in Leisure‐Time Running? Evidence Versus Beliefs.” Journal of Athletic Training 55, no. 12: 1215–1223. 10.4085/1062-6050-523-19. 33064799 PMC7740063

[ejsc70063-bib-0025] Malisoux, L. , A. Urhausen , N. Flores , D. Theisen , and C. Morio . 2024. “Running Shoe Cushioning Properties at the Rearfoot and Forefoot and Their Relationship to Injury: Study Protocol for a Randomised Controlled Trial on Leisure‐Time Runners.” BMJ Open Sport & Exercise Medicine 10, no. 4: e002217. 10.1136/bmjsem-2024-002217. PMC11481106 39415882

[ejsc70063-bib-0026] Milani, T. L. , E. M. Hennig , and M. A. Lafortune . 1997. “Perceptual and Biomechanical Variables for Running in Identical Shoe Constructions With Varying Midsole Hardness.” Clinical Biomechanics 12, no. 5: 294–300. 10.1016/s0268-0033(97)00008-9. 11415737

[ejsc70063-bib-0027] Moher, D. , S. Hopewell , K. F. Schulz , et al. 2010. “CONSORT 2010 Explanation and Elaboration: Updated Guidelines for Reporting Parallel Group Randomised Trials.” BMJ 340, no. mar23 1: c869. 10.1136/bmj.c869. 20332511 PMC2844943

[ejsc70063-bib-0028] Mohr, M. , C. Meyer , S. Nigg , and B. Nigg . 2017. “The Relationship Between Footwear Comfort and Variability of Running Kinematics.” Supplement, Footwear Science 9, no. s1: S45–S47. 10.1080/19424280.2017.1314329.

[ejsc70063-bib-0029] Napier, C. , and R. W. Willy . 2018. “Logical Fallacies in the Running Shoe Debate: Let the Evidence Guide Prescription.” British Journal of Sports Medicine 52, no. 24: 1552–1553. 10.1136/bjsports-2018-100117. 30352861

[ejsc70063-bib-0030] Nigg, B. M. 2001. “The Role of Impact Forces and Foot Pronation: A New Paradigm.” Clinical Journal of Sport Medicine 11, no. 1: 2–9: [Review]. 10.1097/00042752-200101000-00002. 11176139

[ejsc70063-bib-0031] Nigg, B. M. , J. Baltich , S. Hoerzer , and H. Enders . 2015. “Running Shoes and Running Injuries: Mythbusting and a Proposal for Two New Paradigms: ‘Preferred Movement Path’ and ‘Comfort Filter’.” British Journal of Sports Medicine 49, no. 20: 1290–1294. 10.1136/bjsports-2015-095054. 26221015

[ejsc70063-bib-0032] Saragiotto, B. T. , T. P. Yamato , and A. D. Lopes . 2014. “What Do Recreational Runners Think About Risk Factors for Running Injuries? A Descriptive Study of Their Beliefs and Opinions.” Journal of Orthopaedic & Sports Physical Therapy 44, no. 10: 733–738. 10.2519/jospt.2014.5710. 25155860

[ejsc70063-bib-0033] Tennant, P. W. , E. J. Murray , K. F. Arnold , et al. 2021. “Use of Directed Acyclic Graphs (DAGs) to Identify Confounders in Applied Health Research: Review and Recommendations.” International Journal of Epidemiology 50, no. 2: 620–632. 10.1093/ije/dyaa213. 33330936 PMC8128477

[ejsc70063-bib-0034] Theisen, D. , L. Malisoux , J. Genin , N. Delattre , R. Seil , and A. Urhausen . 2014. “Influence of Midsole Hardness of Standard Cushioned Shoes on Running‐Related Injury Risk.” British Journal of Sports Medicine 48, no. 5: 371–376. 10.1136/bjsports-2013-092613. 24043665

[ejsc70063-bib-0035] Theisen, D. , L. Malisoux , P. Gette , C. Nührenbörger , and A. Urhausen . 2016. “Footwear and Running‐Related Injuries—Running on Faith?” Sports Orthopaedics and Traumatology Sport‐Orthopädie—Sport‐Traumatologie 32, no. 2: 169–176. 10.1016/j.orthtr.2016.03.047.

[ejsc70063-bib-0036] Videbaek, S. , A. M. Bueno , R. O. Nielsen , and S. Rasmussen . 2015. “Incidence of Running‐Related Injuries per 1000 h of Running in Different Types of Runners: A Systematic Review and Meta‐Analysis.” Sports Medicine 45, no. 7: 1017–1026. 10.1007/s40279-015-0333-8. 25951917 PMC4473093

[ejsc70063-bib-0037] Wolthon, A. , R. O. Nielsen , R. W. Willy , J. A. Taylor‐Haas , and M. R. Paquette . 2020. “Running Shoes, Pronation, and Injuries: Do Beliefs of Injury Risk Factors Among Running Shoe Salespersons and Physiotherapy Students Align With Current Aetiology Frameworks?” Footwear Science 12, no. 2: 101–111. 10.1080/19424280.2020.1734869.

[ejsc70063-bib-0038] Yamato, T. P. , B. T. Saragiotto , and A. D. Lopes . 2015. “A Consensus Definition of Running‐Related Injury in Recreational Runners: A Modified Delphi Approach.” Journal of Orthopaedic & Sports Physical Therapy 45, no. 5: 375–380. 10.2519/jospt.2015.5741. 25808527

