# Supplementary material for: Association of Shoe Cushioning Perception and Comfort With Injury Risk in Leisure‐Time Runners: A Secondary Analysis of a Randomised Trial
Source: Eur J Sport Sci. 2025 Oct 22;25(11):e70063. doi: 10.1002/ejsc.70063 (PMC12541242; doi:10.1002/ejsc.70063)
Supplement: Supplementary file 1 — Supporting Information S1 [file EJSC-25-e70063-s001.docx]

SUPPLEMENTAL MATERIAL

Contents

[**Supplemental Material S1:** Directed Acyclic Graph (DAG) representing the causal pathways between shoe cushioning perception and running-related injury (RRI). 2](#_Toc207010455)

[**Supplemental Material S2:** Flow chart 3](#_Toc207010456)

[**Supplemental Material S3:** Correlation matrix between shoe perception characteristics (n=527). 4](#_Toc207010457)

[**Supplemental Material S4:** Association between shoe perception characteristics and injury risk (n=527), with the second tertile as the reference group. 5](#_Toc207010458)

[**Supplemental Material S5:** Association between shoe perception characteristics (as continuous variables) and injury risk (n=527). 6](#_Toc207010459)

[**Supplemental Material S6:** Association between shoe perception characteristics and injury risk (n=527) stratified by shoe version, with the first tertile as the reference group. 7](#_Toc207010460)

[**Supplemental Material S7:** Comparison of the descriptive statistics between the participants of the parent trial that were included and those excluded. 8](#_Toc207010461)

## **Supplemental Material S1:** Directed Acyclic Graph (DAG) representing the causal pathways between shoe cushioning perception and running-related injury (RRI).

Created on http://dagitty.net. The outcome variable is identified as a blue oval with black outline and the main exposure is identified as green ovals with black outline. Determinants of the exposure are identified as green ovals and potential confounders as red ovals.

**
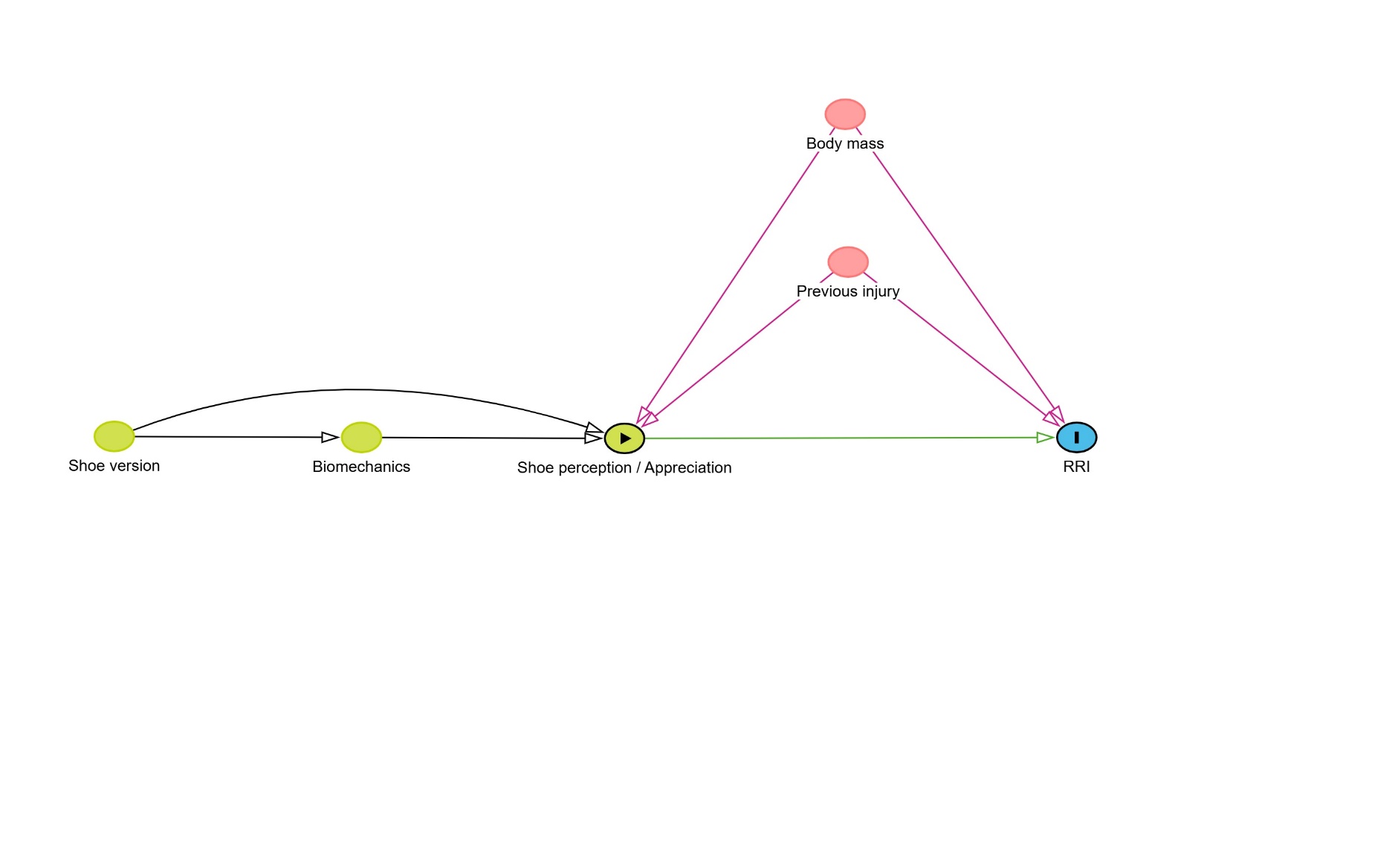
**

## **Supplemental Material S2:** Flow chart


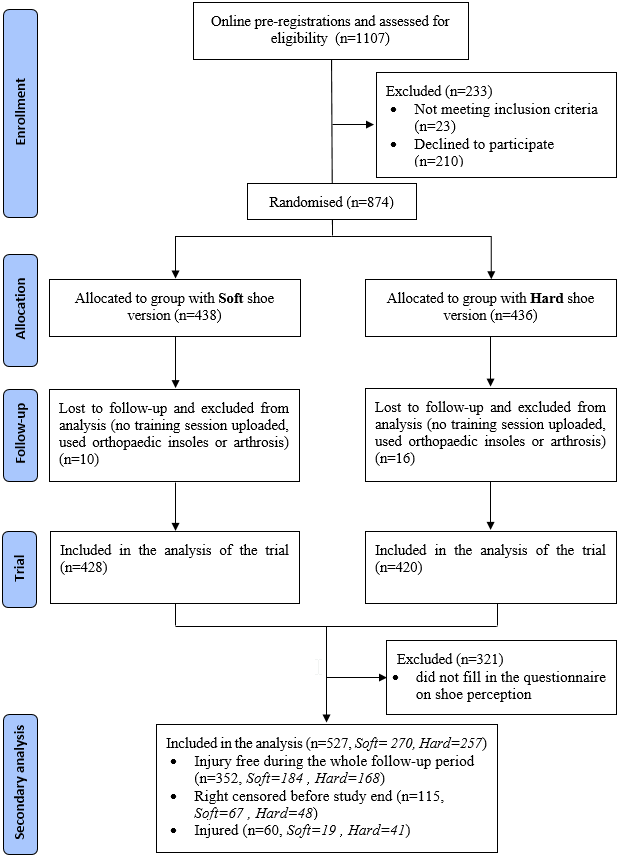


## **Supplemental Material S3:** Correlation matrix between shoe perception characteristics (n=527).

|  | Shoe cushioning perception | Ideal shoe cushioning level | Perceived-ideal cushioning difference | Global appreciation |
| --- | --- | --- | --- | --- |
| Shoe cushioning perception | 1.00 |  |  |  |
|  |  |  |  |  |
| Ideal shoe cushioning level | 0.38 | 1.00 |  |  |
|  | ***<0.001*** |  |  |  |
| Perceived-ideal cushioning difference | 0.57 | -0.48 | 1.00 |  |
|  | ***<0.001*** | ***<0.001*** |  |  |
| Global appreciation | 0.24 | 0.04 | 0.22 | 1.00 |
|  | ***<0.001*** | *0.548* | ***<0.001*** |  |

P-values are presented in *Italic*; P-values <0.05 are presented in Bold.

## **Supplemental Material S4:** Association between shoe perception characteristics and injury risk (n=527), with the second tertile as the reference group.

| Variables | Injuries (participants) | Model 1 | Model 2 | Model 3 |
| --- | --- | --- | --- | --- |
| *Shoe cushioning perception* |  |  |  |  |
| Tertile 1 | 42 (220) | 2.94 [1.56; 5.53]; ***0.001*** | 2.84 [1.51; 5.37]; ***0.001*** | 2.79 [1.44; 5.41]; ***0.002*** |
| Tertile 2 | 12 (181) | Ref | Ref | Ref |
| Tertile 3 | 6 (126) | 0.69 [0.26; 1.84]; *0.459* | 0.69 [0.26; 1.84]; *0.454* | 0.64 [0.24; 1.71]; *0.372* |
| *Ideal shoe cushioning level* |  |  |  |  |
| Tertile 1 | 17 (133) | 1.15 [0.62; 2.14]; *0.648* | 1.13 [0.61; 2.09]; *0.703* | 0.95 [0.51; 1.78]; *0.869* |
| Tertile 2 | 26 (242) | Ref | Ref | Ref |
| Tertile 3 | 17 (152) | 1.09 [0.60; 2.00]; *0.773* | 1.09 [0.59; 2.01]; *0.782* | 1.42 [0.77; 2.61]; *0.258* |
| *Perceived-ideal cushioning diff.* |  |  |  |  |
| Tertile 1 | 32 (166) | 2.44 [1.38; 4.31]; ***0.002*** | 2.28 [1.28; 4.06]; ***0.005*** | - |
| Tertile 2 | 18 (212) | Ref | Ref | - |
| Tertile 3 | 10 (149) | 0.79 [0.36; 1.72]; *0.546* | 0.75 [0.34; 1.64]; *0.470* | - |
| *Global appreciation* |  |  |  |  |
| Tertile 1 | 26 (151) | 1.69 [0.90; 3.18]; *0.101* | 1.68 [0.88; 3.24]; *0.114* | 1.31 [0.66; 2.57]; *0.440* |
| Tertile 2 | 15 (141) | Ref | Ref | Ref |
| Tertile 3 | 19 (235) | 0.78 [0.40; 1.53]; *0.469* | 0.79 [0.40; 1.57]; *0.506* | 0.78 [0.39; 1.56]; *0.483* |

Running exposure: 17,626 hours; 60 running-related injuries; Values are sub-hazard rate ratio [95% confidence interval]; P-values are presented in *Italics*; P-values <0.05 are presented in Bold.

Model 1 is the unadjusted model.

Model 2 = Model 1 adjusted for previous injury and body mass.

Model 3 = Model 2 mutually adjusted for all shoe perception characteristics.

## **Supplemental Material S5:** Association between shoe perception characteristics (as continuous variables) and injury risk (n=527).

| Variables | Model 1 | Model 2 | Model 3 |
| --- | --- | --- | --- |
| Shoe cushioning perception | 0.73 [0.63; 0.85]; ***<0.001*** | 0.78 [0.66; 0.91]; ***0.002*** | 0.72 [0.62; 0.84]; ***<0.001*** |
| Ideal shoe cushioning level | 1.00 [0.86; 1.17]; *0.985* | 1.04 [0.88; 1.22]; *0.680* | 1.13 [0.95; 1.33]; *0.162* |
| Perceived-ideal cushioning diff. | 0.77 [0.68; 0.88]; ***<0.001*** | 0.81 [0.71; 0.92]; ***0.001*** | - |
| Global appreciation | 0.87 [0.78; 0.97]; ***0.016*** | 0.90 [0.80; 1.00]; *0.058* | 0.94 [0.84; 1.04]; *0.239* |

Running exposure: 17,626 hours; 60 running-related injuries; Values are sub-hazard rate ratio [95% confidence interval]; P-values are presented in *Italics*; P-values <0.05 are presented in Bold.

Model 1 is the unadjusted model.

Model 2 = Model 1 adjusted for previous injury and body mass.

Model 3 = Model 2 mutually adjusted for all shoe perception characteristics.

## **Supplemental Material S6:** Association between shoe perception characteristics and injury risk (n=527) stratified by shoe version, with the first tertile as the reference group.

| Variables | Soft shoe version (n=270) | | Hard shoe version (n=257) | |
| --- | --- | --- | --- | --- |
|  | Injuries (participants) | Model 4 | Injuries (participants) | Model 4 |
| *Shoe cushioning perception* |  |  |  |  |
| Tertile 1 | 11 (79) | Ref | 31 (141) | Ref |
| Tertile 2 | 3 (99) | 0.21 [0.06; 0.73]; ***0.014*** | 9 (82) | 0.54 [0.28; 1.14]; *0.107* |
| Tertile 3 | 5 (92) | 0.37 [0.13; 1.08]; *0.069* | 1 (34) | 0.15 [0.02; 1.11]; *0.063* |
| *Ideal shoe cushioning level* |  |  |  |  |
| Tertile 1 | 5 (61) | Ref | 12 (72) | Ref |
| Tertile 2 | 8 (117) | 0.89 [0.29; 2.77]; *0.847* | 18 (125) | 0.91 [0.43; 1.89]; *0.792* |
| Tertile 3 | 6 (92) | 0.83 [0.25; 2.78]; *0.761* | 11 (60) | 1.43 [0.61; 3.33]; *0.409* |
| *Perceived-ideal cushioning diff.* |  |  |  |  |
| Tertile 1 | 7 (61) | Ref | 25 (105) | Ref |
| Tertile 2 | 9 (115) | 0.65 [0.24; 1.75]; *0.400* | 9 (97) | 0.40 [0.19; 0.86]; ***0.019*** |
| Tertile 3 | 3 (94) | 0.25 [0.06; 0.99]; ***0.049*** | 7 (55) | 0.56 [0.24; 1.30]; *0.175* |
| *Global appreciation* |  |  |  |  |
| Tertile 1 | 5 (63) | Ref | 21 (88) | Ref |
| Tertile 2 | 5 (71) | 0.83 [0.24; 2.84]; *0.762* | 10 (70) | 0.60 [0.26; 1.38]; *0.232* |
| Tertile 3 | 9 (136) | 0.34 [0.29; 2.46]; *0.749* | 10 (99) | 0.42 [0.20; 0.91]; ***0.027*** |

Soft shoe version: 19 injuries; Hard shoe version: 41 injuries; Running exposure: 9,578 and 8,048 hours in Soft and Hard shoe groups, respectively; Values are sub-hazard rate ratio [95% confidence interval]; P-values are presented in *Italics*; P-values <0.05 are presented in Bold.

Model 4 = Model 2 (i.e., adjusted for previous injury and body mass) stratified by shoe version.

## **Supplemental Material S7:** Comparison of the descriptive statistics between the participants of the parent trial that were included and those excluded.

| Characteristics | Unit/Qualifier | Included participants  n=527 | Excluded participants  n=321 | p-value |
| --- | --- | --- | --- | --- |
| *Participants’ characteristics* |  |  |  |  |
| Age | Years | 41.1±10.2 | 39.4±9.7 | **0.018** |
| Sex | Male | 341 (64.7%) | 178 (55.5%) | **0.007** |
|  | Female | 186 (35.3%) | 143 (44.5%) |  |
| Height | cm | 174±9 | 173±9 | **0.009** |
| Body mass | kg | 74.3±12.6 | 71.7±12.4 | **0.004** |
| Body mass index | kg.m^-2^ | 24.3±3.1 | 23.9±3.0 | 0.055 |
| Proportion of fat mass | % | 22.5±7.3 | 22.7±7.4 | 0.698 |
| Previous Injury | No | 443 (84.1%) | 268 (83.5%) | 0.826 |
|  | Yes | 84 (15.9%) | 53 (16.5%) |  |
| Running experience | Years | 6 [3-14] | 6 [3-12] | 0.452 |
| Regularity (last 12 months) | Months | 12 [6-12] | 12 [5-12] | 0.413 |
| *Running participation (follow-up)* |  |  |  |  |
| Running frequency | Sessions.week^-1^ | 1.4 [1.0-2.0] | 1.3 [0.9-1.9] | **0.026** |
| Mean session duration | Minutes | 51 [41-61] | 49 [38-60] | **0.045** |
| Mean session distance | km | 8.2 [6.2-10.1] | 7.7 [5.6-9.9] | **0.029** |
| Mean session intensity | a.u. [1-10] | 3.9 [3.9-4.5] | 3.5 [3.0-4.3] | **0.001** |
| Mean speed | km.h^-1^ | 9.7 [8.8-10.7] | 9.6 [8.7-10.5] | 0.438 |

T-test was used for normally distributed continuous variables; Mann-Whitney U test was used for non-normally distributed continuous variables; Chi-squared test was used for categorical variables.
